# Mutation signatures inform the natural host of SARS-CoV-2

**DOI:** 10.1093/nsr/nwab220

**Published:** 2021-12-04

**Authors:** Shanjun Deng, Ke Xing, Xionglei He

**Affiliations:** State Key Laboratory of Biocontrol, School of Life Sciences, Sun Yat-Sen University, China; School of Life Sciences, Guangzhou University, China; State Key Laboratory of Biocontrol, School of Life Sciences, Sun Yat-Sen University, China

The origin of SARS-CoV-2, the causative virus of COVID-19, has been a mystery since the beginning of the outbreak and has been heavily debated lately. One of the main reasons for this is that RaTG13, the closest relative found to date [1] (in the horseshoe bat *Rhinolophus affinis*), has only 96.1% nucleotide similarity to SARS-CoV-2 (with ∼1200 nucleotide differences). The situation is distinct from the two previous coronavirus outbreaks that happened this century (SARS in 2003 and MERS in 2012); in both cases, a closely related virus with over 99% nucleotide similarity to the causative virus was found in wild animals shortly after the start of each outbreak [2,3]. The missing intermediates between RaTG13 and SARS-CoV-2 prevent a better understanding of the spillover. Fortunately, since the viral mutation spectrum is expected to be heavily shaped by host factors, signatures left on the available viral genomes would inform the pre-outbreak history of SARS-CoV-2.

We included SARS-CoV-2 and six related viruses in the analysis (Fig. 1a). The six related viruses were chosen because they are evolutionarily close enough for reliable mutation inferences while distant enough for observing plenty of mutations. At least three different hosts, bats, pangolins and humans, are involved, highlighting the complex host history of this viral lineage [4,5]. Two separate phylogenetic trees were constructed to avoid the phylogeny confusions caused by recombination (Fig. S1), which resulted in different genealogical histories at different genomic regions in the ancestor of Bat-Cov-ZXC21 and Bat-Cov-ZC45 (both found in the horseshoe bat *Rhinolophus sinicus* [6]). Branch X, which represents the pre-outbreak history of SARS-CoV-2, and B1, which represents the history of RaTG13 after it split from SARS-CoV-2, are present in both phylogenetic trees. Using conventional molecular evolutionary methods [7], we compared the viral genomes to infer the substitution mutations that occurred on the evolutionary branches, as marked in Fig. 1a (See Methods in supplementary data). We considered only the third codon positions so that the obtained mutation spectra were less shaped by selection [8] (Fig. 1b and Table S1). Because the mutations on different evolutionary branches occurred independently, the derived mutation spectra of the branches are independent. To quantify the similarity between two mutation spectra we computed an identity score (i-score), which is the proportion of the total rate variation explained by the x = y dimension in a two-dimensional plot of the two spectra, as in Fig. 1c (Methods). An i-score equal to 100% means the two mutation spectra are 100% identical.

**Figure 1.**
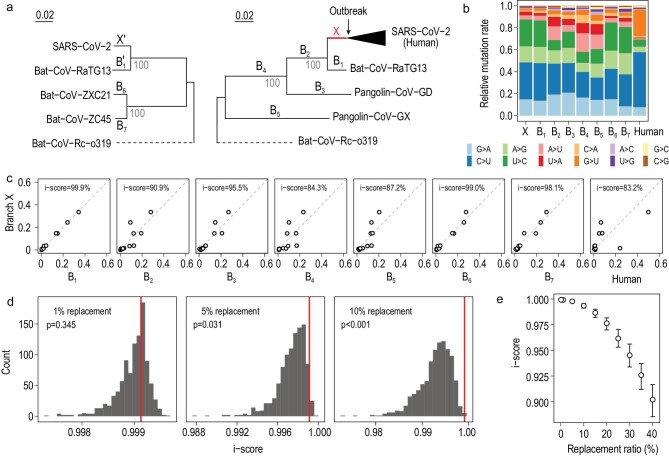
Evolution of the mutation spectrum in the SARS-CoV-2 lineage. (a) The phylogenetic relationships of the seven coronaviruses included in the analysis. Two separate phylogenetic trees are considered to resolve the confusions caused by recombination, which results in different genealogical histories at different genomic regions in the ancestral branch of Bat-CoV-ZXC21 and Bat-CoV-ZC45. Nine major evolutionary branches examined in this study, X, B1–B7 and the human branch, are shown. Branch X and B1 are also present (as X^′^ and B1^′^) in the tree with B6 and B7 to help infer the ancestor of B6 and B7. Bat-CoV-Rc-o319 is used as an outgroup in both trees. (b) The relative mutation rate of the 12 mutation types on each of the nine evolutionary branches. (c) The similarity of mutation spectra between branch X and each of the other eight branches. The similarity of two branches is measured by identity score (i-score), which is the proportion of total rate variation explained by the x = y dimension in the plot of the two spectra. (d) The sensitivity of i-score between branch X and B1 to potential perturbations on X. Each histogram represents the result of 1000 replicates. The rate of replacements by random mutations is shown in each panel, with the red line showing the original i-score between X and B1, and the p showing the frequency of cases with an i-score larger than the original i-score. (e) The sensitivity of i-score under different replacement rates. The hollow point represents the median of 1000 replicates, and the error bar covers the upper and lower quartiles.

The mutation spectra calculated separately in the two phylogenetic trees are nearly identical for the same branches (i-score = 99.9% for X versus X^′^ and 99.4% for B1 versus B1^′^; Fig. S2), suggesting that the results of the two trees are comparable. There are three notable features regarding the obtained spectra (Fig. 1b and c). First, branch X is nearly identical to B1, with an i-score of 99.9%. To assess how the signal is sensitive to potential perturbation we replaced a certain proportion of the mutations on branch X with random mutations (Methods). We observed a rapid reduction of i-score as a function of the replacement rate (Fig. 1d and e). Second, branch X is distinct from the post-outbreak branch of SARS-CoV-2 (i.e. the human branch), with an i-score of 83.9%. Compared to branch X, the human branch has a lot more G > U and C > U mutations, suggesting much stronger mutational pressures imposed by the reactive oxygen species (ROS) and APOBEC family, respectively, on the SARS-CoV-2 genome in infected human cells. Meanwhile, the rates of A > G/U > C mutations reduce substantially, suggesting weaker activity of the ADAR family. Third, branch X is in general highly similar to the branches that have bats as the putative hosts (B1, B6 and B7) while less similar to the branches with non-bat hosts involved. These results, in particular the 99.9% similarity between X and B1, suggest SARS-CoV-2 was not artificially synthesized for gain-of-function research, because the mutation spectrum is of little functional relevance and a synthesized genome is unlikely to show a similar mutation spectrum to a naturally evolved viral genome (RaTG13). Notably, making similar mutation spectra is doable by nature for close sister lineages like B6 and B7 (Fig. S2).

The viral mutations are caused by both replication errors and replication-independent lesions or editing. The former is mostly associated with the viral self-encoded replication-transcription complex (RTC) and the latter would be mostly explained by host factors [9] (Fig. 2a). The same type of replication errors occurred in the synthesis of positive-sense strand or negative-sense strand would result in different types of mutation. For example, the same replication error of, say, C-to-A, in the C-to-G step would cause a C > U mutation in the replication of C but a G > A mutation in the replication of G (Fig. 2a). Other types of replication errors have the same feature, resulting in each complementary pair formed by 12 mutation types having the same rate if all mutations were due to replication errors. In contrast, the different mutation rate observed in each complementary pair would be ascribed to replication-independent factors, which are associated in large part with the host.

To obtain the host signatures we calculated the rate difference in each complementary pair. The six host signatures (S1–S6), each corresponding to a complementary pair, are indeed informative (Fig. 2b). For example, S1, the rate of C > U minus the rate of G > A, ranges from 0.06 to 0.42 among the different evolutionary branches. This may represent the different activities of the APOBEC family in different hosts. S2, the rate of U > C minus the rate of A > G, ranges from −0.03 to 0.1. This is likely associated with the relative activity of the ADAR family. S3, the rate of G > U minus the rate of C > A, ranges from −0.03 to 0.23 and appeared unusually strong in the human branch. This could be related to This could be related to ROS that preferentially target the single-stranded RNA [7] and have a strong induction in the infected human cells. Notably, the mentioned genes/pathways are just putatively associated with the observed host signatures. We found that branch X has nearly identical host signatures to B1, with an i-score of 99.5%, despite substantial deviations from the human-

or pangolin-associated branches (Fig. 2c). A multidimensional scaling plot shows that X is almost perfectly overlapping with B1, close to B6 and B7, and distant from the other branches (Fig. 2d). To eliminate concerns about the quality of the assembled genome of RaTG13, we reproduced the above analyses and found largely the same results after replacing RaTG13 with other bat coronaviruses, RshSTT182 and BANAL-52, with 92.6% and 96.8% genomic similarity to SARS-CoV-2 respectively (Figs S3–S6). These results suggest that SARS-CoV-2 shared almost the same host environment with RaTG13 before the outbreak, which is consistent with the previous study [10].

**Figure 2.**
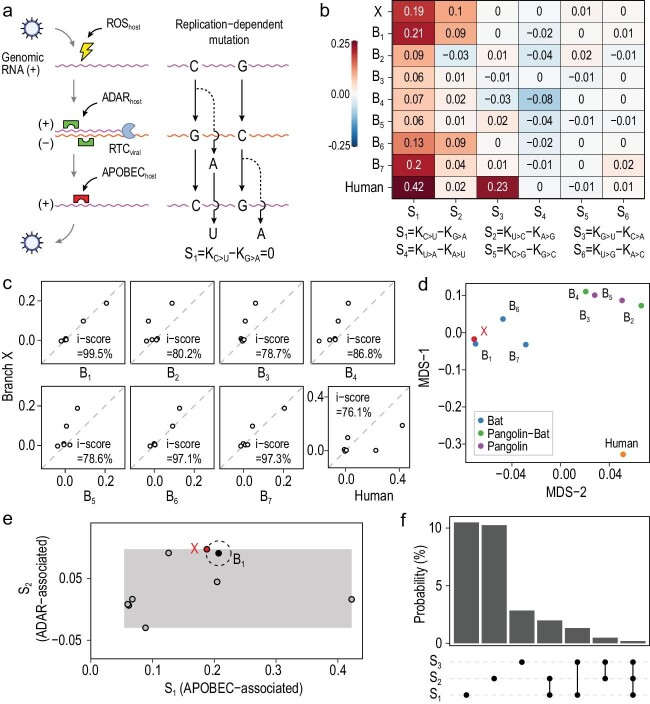
Host signatures inferred from the viral mutation spectrum. (a) A diagram showing the major sources of viral mutations, which include the replication errors (by the viral replication-transcription complex, RTC) and the lesions caused by host factors. Because replication processes are the same, despite being in the opposite order, for nucleotides G and C (or A and T), replication errors would result in equal rates of complementary mutations such as C > A and G > T. However, host factors would distort the equal-rate pattern of complementary mutation pairs. The positive-sense RNA is often in a single-stranded form, sensitive to ROS and the APOBEC family, while the negative-sense RNA tends to be in a double-stranded form, thus more affected by the ADAR family. (b) The rate difference of each complementary mutation pair serves as a signature of host factors. There are thus six host signatures, each corresponding to a complementary mutation pair, inferred from the viral mutation spectrum. Among the three major host signatures, S1 is likely associated with the APOBEC family, S2 the ADAR family and S3 the ROS. (c) The similarity of host signatures between branch X and each of the other eight branches. Branch X is highly similar to B1, B6 and B7, the three branches of bat coronavirus. (d) A multidimensional scaling (MDS) plot of the host signatures reveals that branch X and B1 have nearly the same positions. (e) Estimation of the likelihood that an arbitrary laboratory condition happens to match the host signatures of B1 (the branch of RaTG13). The grey rectangular area is defined by the empirical ranges of S1 (APOBEC-associated) and S2 (ADAR-associated) that are based on the data of panel (b). The probability of approaching B1 as closely as X is the area of the circle divided by the whole rectangular area, which is ∼2.0%. The positions of the other seven branches are also shown in the rectangular area. (f) The probability that an arbitrary condition approaches B1 as closely as X is given, by considering the different combinations of S1, S2 and S3, respectively.

To gauge the probability of an arbitrary cell culture condition in the laboratory matching the natural host environment of RaTG13, we estimated the size of the space formed by the host signatures, each of which has an empirical range corresponding to the nine branches presented in Fig. 2b (Methods). As shown in Fig. 2e, the probability of approaching branch B1 as closely as SARS-CoV-2 is ∼2.0%, if S1 and S2 are considered. The number would be 0.02% if S3 is also considered (Fig. 2f). The estimations are conservative as we only considered S1, S2 and S3, which have the largest empirical ranges and apparently independent associated genes/pathways (APOBEC, ADAR and ROS). We caution the calculations that assumed the associated gene/pathway activities are uniformly distributed within the empirical ranges. Nevertheless, the results are helpful when thinking of the likelihood that an arbitrary cell culture condition set in a laboratory would happen to duplicate a defined natural host environment.

It should be emphasized that this study addresses the evolution of the SARS-CoV-2 genome but nothing else. Using mutational signatures inferred by the available viral genomes we probed the evolutionary window of time (branch X) that SARS-CoV-2 had before the outbreak and after the split from bat coronavirus RaTG13. The missing intermediates within this time window, that presumably span tens of years [11], prevent a better understanding of the spillover. Our analyses, based on public data, provide compelling evidence that during this time window SARS-CoV-2 evolved in a host environment highly similar, if not identical, to other five bat coronaviruses (BANAL-52, RaTG13, RshSTT182, ZXC21 and ZC45). One may argue that, while branch X as a whole is compatible with natural laws, it may not be at a few key sites. Such an argument presumes that there are intermediates with over 99% similarity to SARS-CoV-2 to be found in nature. Notably, claiming such natural intermediates would leave little room for speculation, as in the cases of SARS [2] and MERS [3]. The mission of the scientific community, then, is to find them in nature to better understand the spillover.

## Supplementary Material

nwab220_Supplemental_Files
