# Supplementary material for: Mutation signatures inform the natural host of SARS-CoV-2
Source: Natl Sci Rev. 2021 Dec 4;9(2):nwab220. doi: 10.1093/nsr/nwab220 (PMC8690307; doi:10.1093/nsr/nwab220)
Supplement: nwab220_Supplemental_Files [file nwab220_supplemental_files.zip › SI.pdf]

Supplementary Information of

"Mutation signatures inform the natural host of SARS-CoV-2"

Shanjun Deng[1,#], Ke Xing[2,#], & Xionglei He[1]

[1]State Key Laboratory of Biocontrol, School of Life Sciences, Sun Yat-sen University, Guangzhou 510275, China

[2]School of Life Sciences, Guangzhou University, Guangzhou 511442, China

[#]These authors contributed equally.

Correspondence should be addressed to X. H. (hexiongl@mail.sysu.edu.cn)

This file contains:

Appendix

Methods

Legends of Supplementary Datasets I-VII

Supplementary Figures 1-6

Supplementary Tables 1-3

Data availability

Code availability

Appendix

When this work appeared online as a preprint, a related paper from an independent group with a similar conclusion was published[11].

Methods

Genomic Data

The SARS-CoV-2 related bat and pangolin coronavirus genomic sequences were obtained from NCBI GenBank (https://www.ncbi.nlm.nih.gov/genbank). For genomes without accurate annotations of ORFs, we re-annotated these genomes with CDSs annotated in SARS-CoV-2 by Exonerate2 (–model protein2genome: bestfit –score 5 -g y)[12]. The complete genomic sequences and metadata of SARS-CoV-2 were retrieved from Global Initiative on Sharing All Influenza Data (GISAID; https://www.gisaid.org/; accessed on 19 March 2021)[13]. Gap-containing genomes in examined regions were removed, and only genomes from Dec. 2019 to Dec. 2020 were chosen for analysis. All available genomes submitted to GISAID from Dec. 2019 to Feb. 2020 were included, and, among the too many submitted genomes from Mar. to Dec. 2020, 2,000 genomes were randomly selected for each month. Finally, a total of 21,432 SARS-CoV-2 genomes were included. Following GISAID we used SARS-CoV-2 WIV04 (EPI_ISL_402124) as the reference genome to process human genomes. The detailed information of SARS-CoV-2 and the related coronaviruses included in this analysis is summarized in Supplementary Dataset I.

Phylogenetic analysis and mutation spectra calculation

The codon alignments of ORFs were performed based on amino acid sequences translated by TranslatorX[14] and MAFFT v7.471 (mafft --inputorder --adjustdirection --auto)[15], and further concatenated by AMAS[16]. Only ORFs with consistent annotations in the examined viruses were included. Maximum likelihood phylogenetic analysis based on the whole coding regions was conducted by using IQ-TREE v2.0.3[17] with GTR+FO+R10 substitution model and 1,000 bootstrap replicates. The ancestral sequences of the internal nodes were inferred in IQ-TREE with an *-asr* parameter, and mutations on each branch were derived by comparing the ancestral sequence to the descendant sequence. To avoid the confounding effects of potential recombination and convergent evolution, the region covering the receptor binding domain and the furin-like cleavage site (330th-790th codons on genome of SARS-CoV-2) of the spike protein was removed from the analysis. Only the third codon positions were considered in calculation of the mutation spectra. The aligned sequences can be found in Supplementary Dataset II-VII.

To obtain the after-outbreak mutations of SARS-CoV-2, 59 separate main clades each containing more than 100 sequences and supported by a bootstrap value >90 were selected from the phylogenic tree. Mutations were inferred by comparing each individual sequences to the corresponding common ancestral sequences of each clade, respectively. To avoid redundancy, recurrent mutations within a clade were counted

once. Then, the 59 clade-specific ancestral sequences were compared to the earliest common ancestral sequence of SARS-CoV-2. Mutations obtained from the two steps were pooled to derive the mutation spectrum of the Human branch.

For a specific mutation type, say C>A, the rate was calculated as the number of C>A mutations divided by the total number of C nucleotides in the ancestral sequence of the given branch (third codon positions). The mutation rates of the 12 mutation types were then each divided by their sum to obtain the relative mutation rates (i.e., mutation spectrum). The i-score of two mutation spectra is the proportion of variance explained by the x=y dimension in a two-dimensional plot of the two spectra. Specifically, let $A = (S_1, S_2)$, where S_1 and S_2 are the two mutation spectra under examination, and $B = (D_1, D_2)$, where D_1 is the projection of A onto the x=y dimension and D_2 onto the x= -y dimension. Then, the i-score = $var(D_1) / (var(S_1) + var(S_2))$. To assess how the i-score between branch X and B1 is sensitive to potential perturbation we replaced a certain number (*n*) of the mutations on branch X with random mutations. Specifically, a random set of *n* mutations on branch X were deleted and *n* mutations with equal probability in terms of the 12 mutation types were added. The resulting mutation spectrum on X was then used to compute the i-score with B1.

To verify the whole-genome-based evolutionary branches at different genomic regions a sliding window analysis through the viral genomes was conducted. Specifically, each window covers 500 codons (or 1,500 nucleotides, ~5% of the viral genome) and the step size is a half window. For each window we constructed the

phylogeny of the viruses using synonymous sites, and then checked if the whole-genome-based branches exist in the window. Neighbor-Joining phylogeny was obtained in MEGA X[18], which allows such analysis on synonymous sties, with 1,000 bootstrap replicates.

To assess the probability that a random condition happens to match the host signatures of B1, for each host signature we used the maximum value and minimum value to define its empirical range. For example, for host signature 1 the maximum value and minimum value are 0.42 and 0.06, respectively. There are three conditions for calculating the probability: First, the probability in one-dimension situation is defined as r divided by l_i, where r is the Euclidean distance between X and B1 under the given condition and l_i is the empirical range of host signature i. Second, considering a two-dimensional space form by signature i and j, the probability is defined as the area of the circle with radius r divided by the multiplication of $\{l_i, l_j\}$. Third, considering a three-dimensional space form by signature i, j and k, the probability is defined as the volume of the ball with radius r divided by the multiplication of $\{l_i, l_j, l_k\}$.

Legends of Supplementary Datasets

The supplementary dataset I has the accession information of the genomes used in this study. The other six datasets (II-VII) contain the codon-based alignments of the genomic sequences of the viruses indicated in each file's name. The files are used to derive the mutations of the evolutionary branches examined in this study.

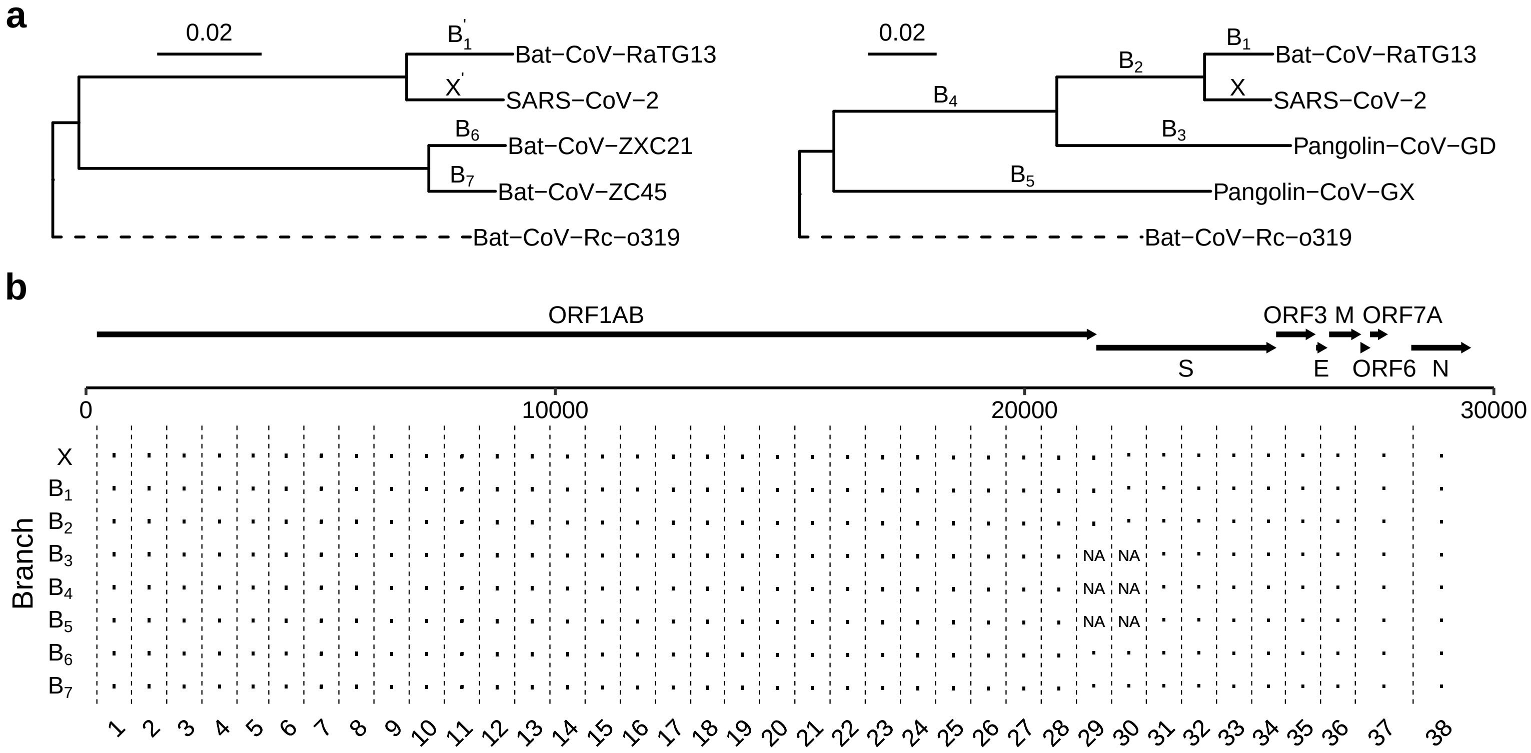

Fig. S1. Verification of the evolutionary branches (X and B1-B7) at different genomic regions. Recombination can distort at some genomic regions the whole-genome-based phylogenetic topology. As a result, some evolutionary branches based on whole genomes may not exist at some genomic regions. To solve the problem, two separate phylogenetic trees are considered. The evolutionary branches marked in the two trees (X and B1-B7) should exist at the vast majority of the genomic regions according to ref. 4. To be rigorous, a sliding window analysis through the viral genomes was conducted to verify the whole-genome-based evolutionary branches at different genomic regions. Specifically, each window covers 500 codons (or 1,500 nucleotides, ~5% of the viral genome) and the step size is a half window. For each window we constructed the phylogeny of the viruses using synonymous sites, and then checked if the whole-genome-based branches exist in the window. **a.** The two phylogenetic trees of the involved viruses based on their whole genome sequences. **b.** There are two regions (#29 and #30, covering nucleotides 21,105-22,593) in which B3, B4, and B5 do not exist (marked as NA), a finding consistent with ref. 4. The two regions are thus not considered in the calculation of the mutation spectrum of B3, B4 and B5. The coordinate of SARS-CoV-2 is used and the open reading frames examined are shown.

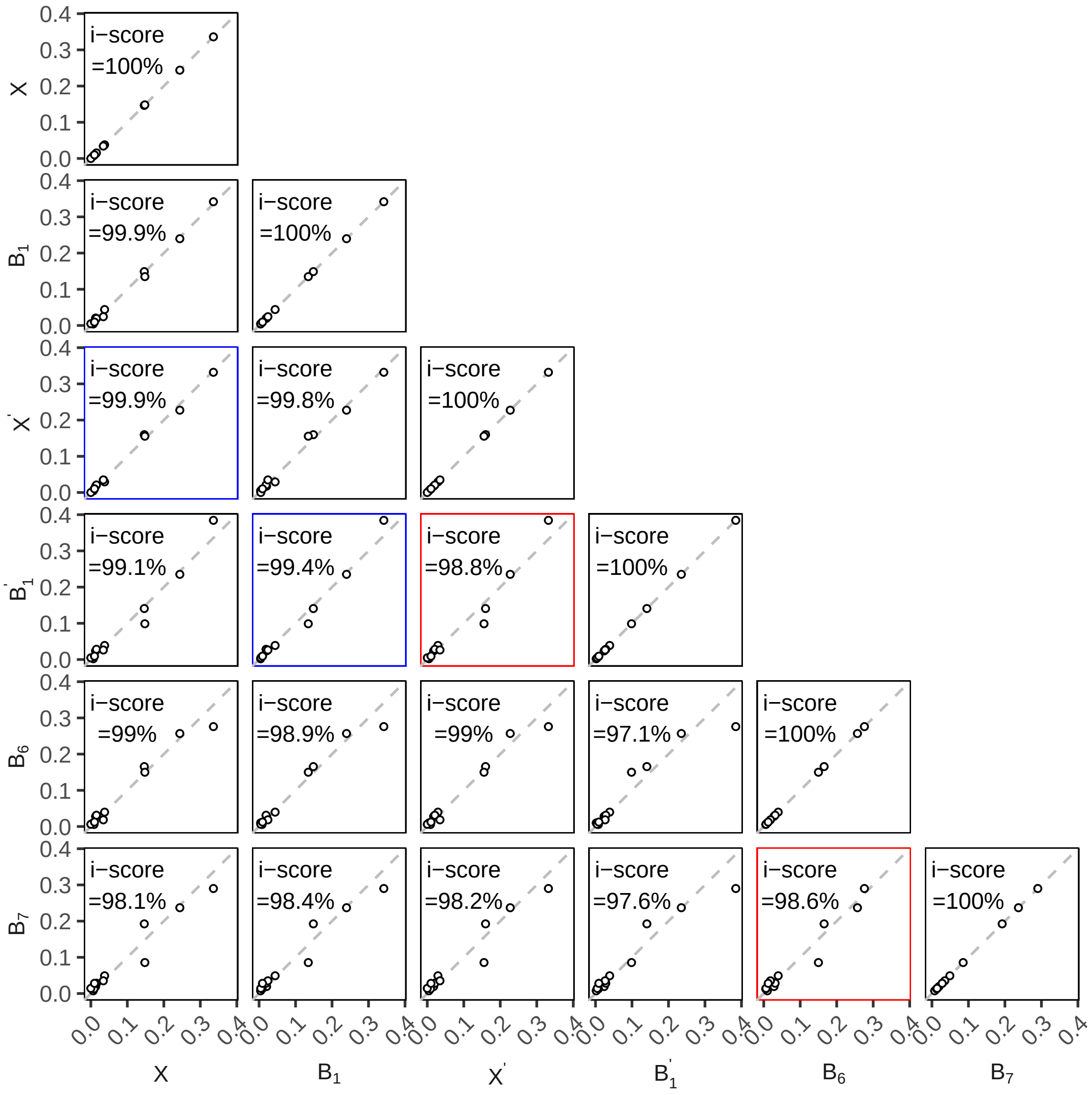

Fig. S2. The pairwise similarities of mutation spectrum among branches X, B1, X', B1', B6 and B7. The similarities between X and X' and between B1 and B1' (highlighted in blue) indicate results from the two separate phylogenies comparable. The i-score of B6 and B7 is similar to that of X' and B1' (highlighted in red). The reduced i-score of X' and B1' relative to that of X and B is likely due to the rather distant outgroups that affect the inference of the ancestor of X' and B1'.

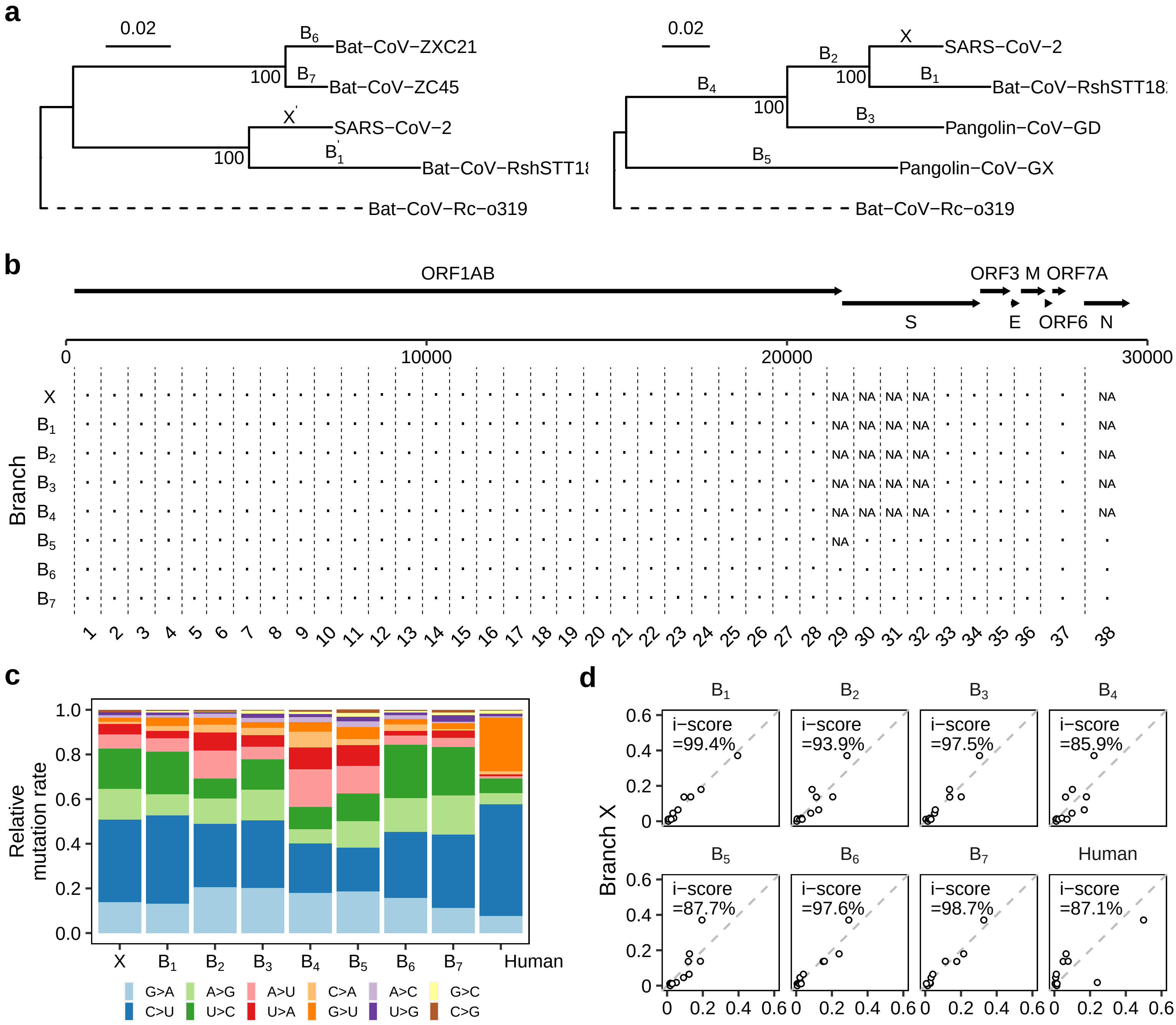

Fig. S3. Mutation spectra after replacing RaTG13 with RshSTT182. a. The two phylogenetic trees of the involved viruses based on their whole genome sequences. **b.** Verification of the whole-genome-based branches (X and B1-B7) at different genomic regions. The regions #29, #30, #31, #32, and #38 (covering nucleotides 21,108-24,087 and 28,267-29,496) are affected by recombination (marked as NA), which is consistent with the ref. 19. They are all excluded from further analyses. The coordinate of SARS-CoV-2 is used and the open reading frames examined are shown. **c.** The relative mutation rate of the 12 mutation types on each of the nine evolutionary branches. **d.** The similarity of mutation spectrum between branch X and each of the other eight branches.

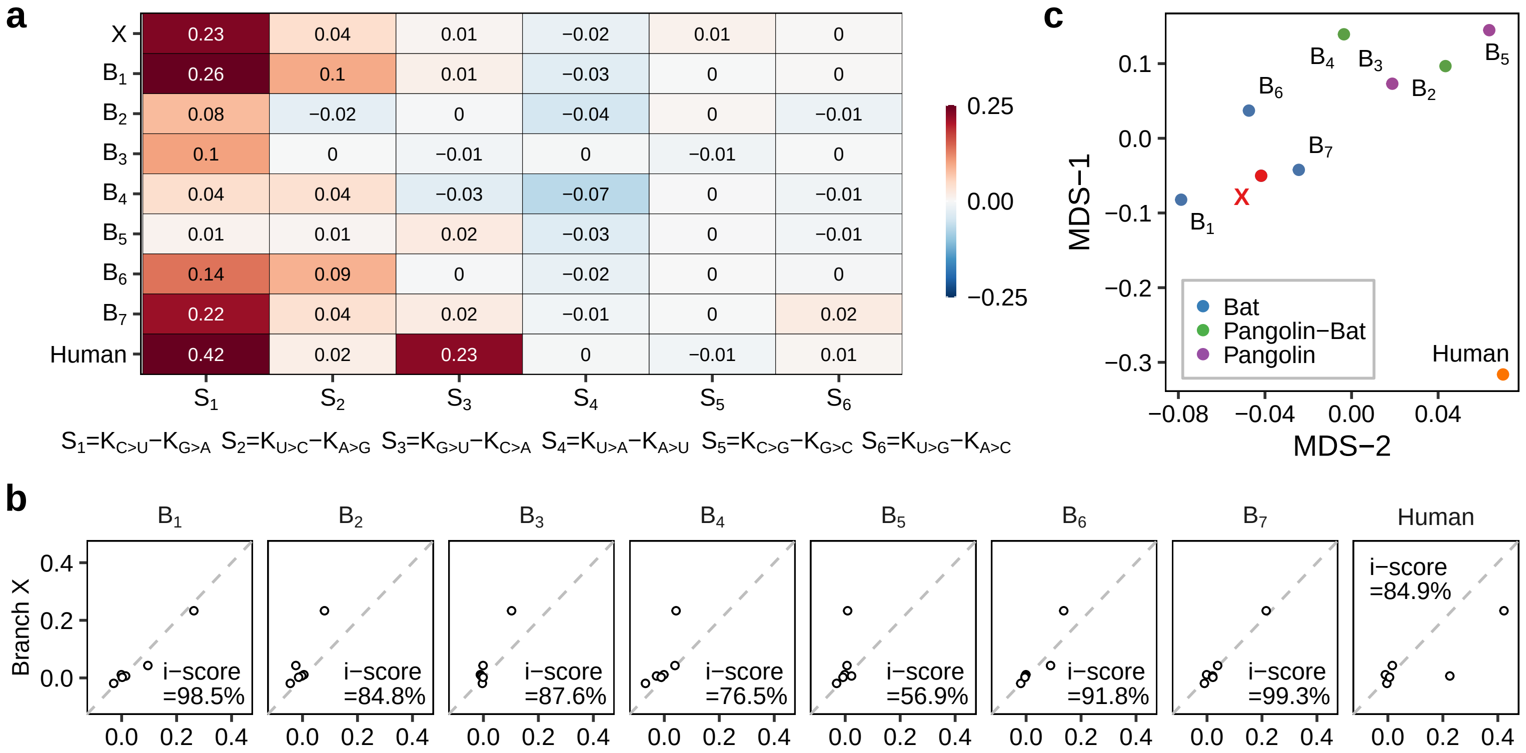

	S_1	S_2	S_3	S_4	S_5	S_6
X	0.23	0.04	0.01	−0.02	0.01	0
B_1	0.26	0.1	0.01	−0.03	0	0
B_2	0.08	−0.02	0	−0.04	0	−0.01
B_3	0.1	0	−0.01	0	−0.01	0
B_4	0.04	0.04	−0.03	−0.07	0	−0.01
B_5	0.01	0.01	0.02	−0.03	0	−0.01
B_6	0.14	0.09	0	−0.02	0	0
B_7	0.22	0.04	0.02	−0.01	0	0.02
Human	0.42	0.02	0.23	0	−0.01	0.01

Fig. S4. Host signatures after replacing RaTG13 with RshSTT182. a. The six host signatures of the nine evolutionary branches. **b.** The similarity of host signatures between branch X and each of the other eight branches. **c.** The MDS plot of the host signatures reveals X is surrounded by the three branches (B1, B6, and B7) with horseshoe bats as hosts.

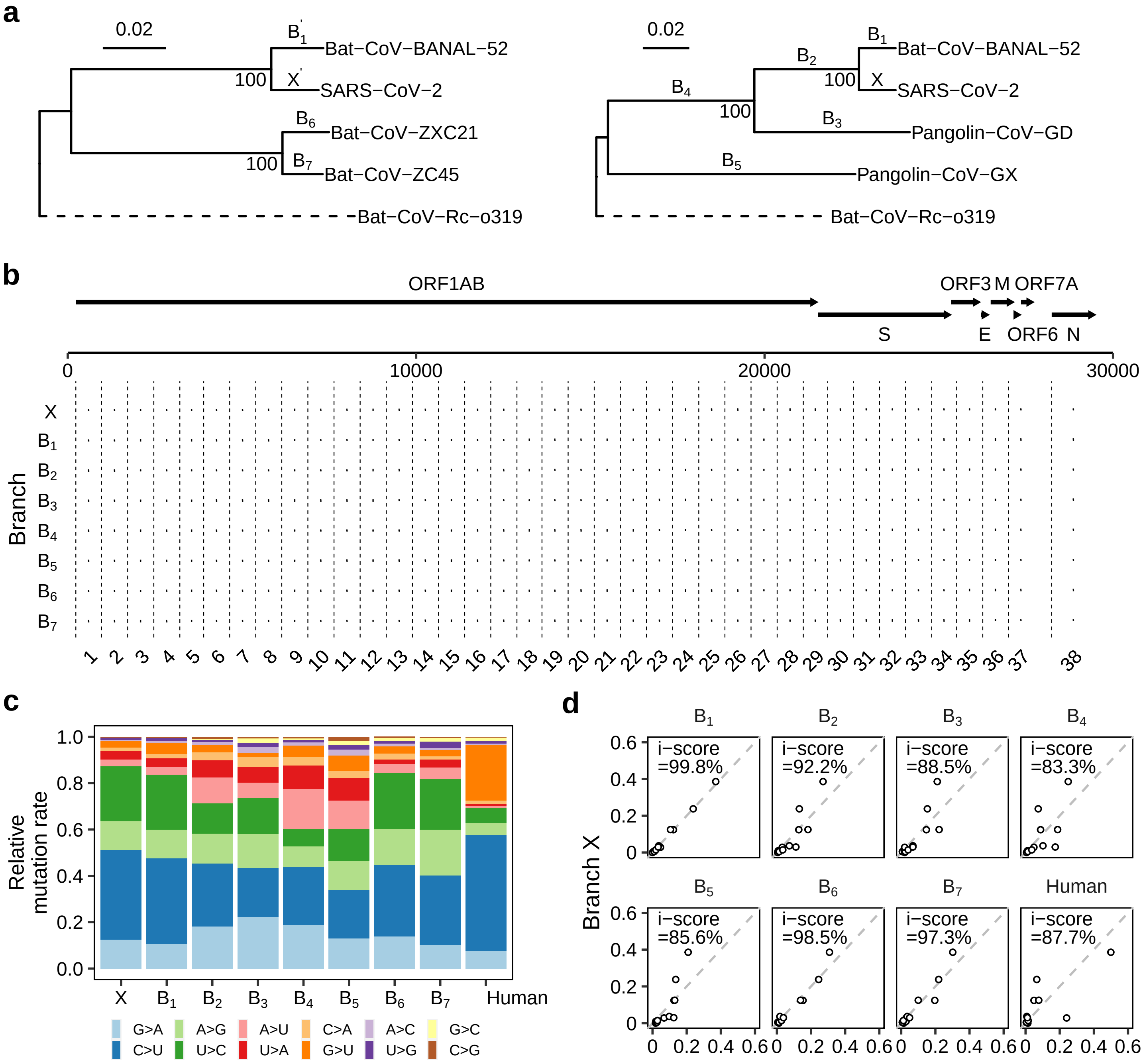

Fig. S5. Mutation spectra after replacing RaTG13 with BANAL-52. a. The two phylogenetic trees of the involved viruses based on their whole genome sequences. **b.** Verification of the whole-genome-based branches (X and B1-B7) at different genomic regions. The regions #30 (covering nucleotides 21,811-22,548) are affected by recombination (marked as NA), which is consistent with the BANAL-52. They are all excluded from further analyses. The coordinate of SARS-CoV-2 is used and the open reading frames examined are shown. **c.** The relative mutation rate of the 12 mutation types on each of the nine evolutionary branches. **d.** The similarity of mutation spectrum between branch X and each of the other eight branches.

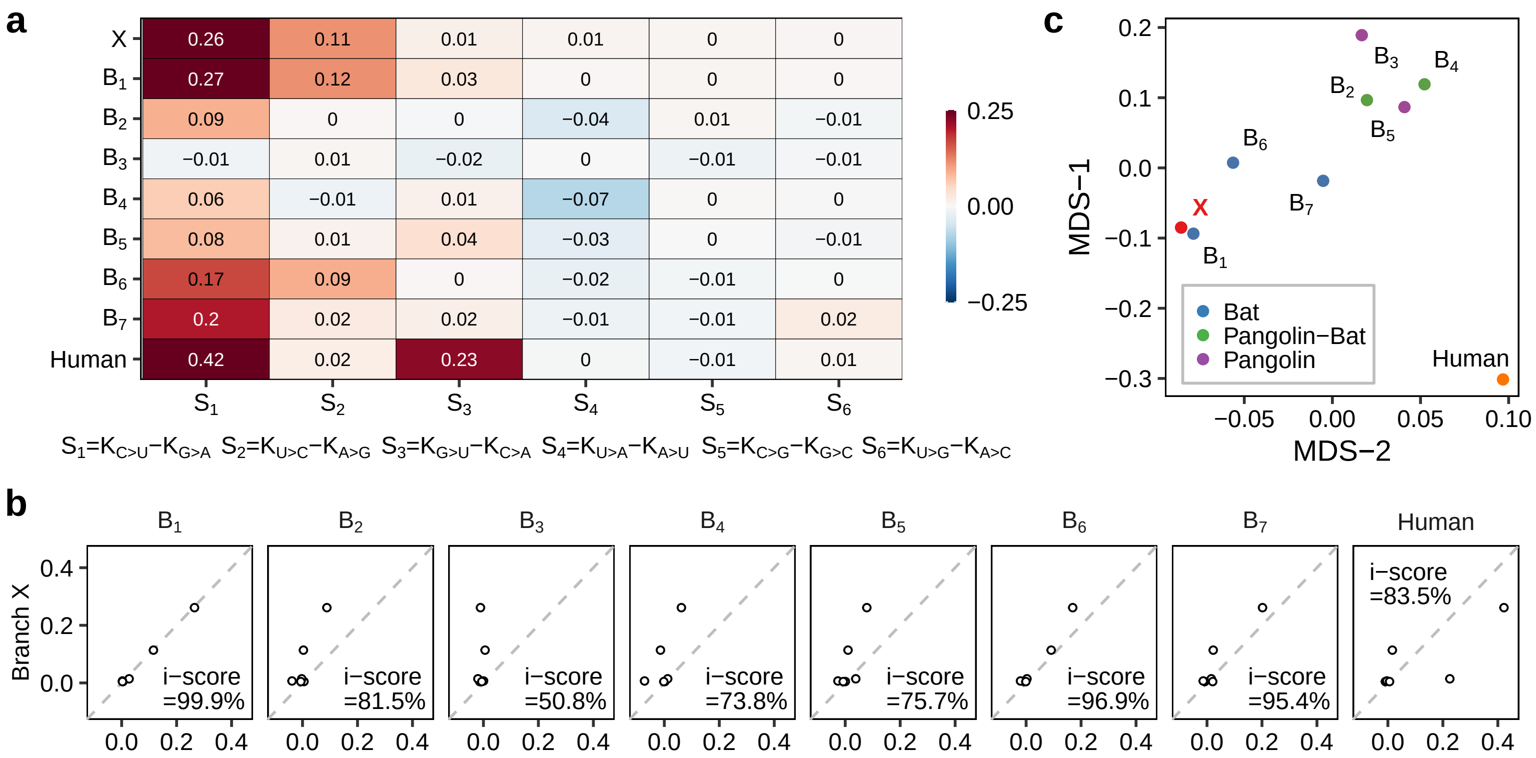

Fig. S6. Host signatures after replacing RaTG13 with BANAL-52. **a.** The six host signatures of the nine evolutionary branches. **b.** The similarity of host signatures between branch X and each of the other eight branches. **c.** The MDS plot of the host signatures reveals X is surrounded by the three branches (B1, B6, and B7) with horseshoe bats as hosts.

Table. S1. Mutation spectrum (RaTG13)

Branch	Mutation	Count_Ref	Count_Alt	RelativeMutationRate
X	A>C	2601	3	0.006657384
X	A>G	2601	66	0.146462449
X	A>T	2601	17	0.037725176
X	C>A	1323	3	0.013088326
X	C>G	1323	2	0.008725551
X	C>T	1323	77	0.335933711
X	G>A	1131	29	0.147998768
X	G>T	1131	3	0.015310217
X	G>C	1131	0	0
X	T>A	4047	24	0.034229515
X	T>C	4047	171	0.243885294
X	T>G	4047	7	0.009983609
B1	A>C	2600	2	0.004191267
B1	A>G	2600	71	0.148789988
B1	A>T	2600	21	0.044008306
B1	C>A	1323	5	0.020592016
B1	C>G	1323	2	0.008236806
B1	C>T	1323	83	0.341827467
B1	G>A	1131	28	0.134891361
B1	G>C	1131	1	0.004817549
B1	G>T	1131	4	0.019270194
B1	T>A	4046	18	0.024240152
B1	T>C	4046	178	0.239708168
B1	T>G	4046	7	0.009426726
B2	A>C	2672	15	0.016862456
B2	A>G	2672	109	0.122533849
B2	A>T	2672	111	0.124782177
B2	C>A	1339	11	0.024676192
B2	C>G	1339	8	0.017946321
B2	C>T	1339	125	0.280411272
B2	G>A	1112	71	0.191787189
B2	G>C	1112	1	0.002701228
B2	G>T	1112	13	0.035115964
B2	T>A	4083	116	0.085338429
B2	T>C	4083	126	0.09269519
B2	T>G	4083	7	0.005149733
B3	A>C	2539	21	0.01733328
B3	A>G	2539	168	0.138666243
B3	A>T	2539	73	0.060253784
B3	C>A	1278	22	0.036075802
B3	C>G	1278	2	0.003279618
B3	C>T	1278	164	0.268928705
B3	G>A	1052	105	0.209169196
B3	G>C	1052	8	0.015936701
B3	G>T	1052	14	0.027889226
B3	T>A	3834	102	0.055753512
B3	T>C	3834	269	0.147036223
B3	T>G	3834	36	0.01967771
B4	A>C	2582	13	0.010599758
B4	A>G	2582	102	0.083167332
B4	A>T	2582	209	0.170411494
B4	C>A	1271	44	0.072881275
B4	C>G	1271	5	0.008281963
B4	C>T	1271	140	0.231894967
B4	G>A	1036	81	0.164601618

Branch	Mutation	Count_Ref	Count_Alt	RelativeMutationRate
B4	G>C	1036	4	0.008128475
B4	G>T	1036	23	0.046738731
B4	T>A	3842	167	0.091509872
B4	T>C	3842	182	0.099729322
B4	T>G	3842	22	0.012055193
B5	A>C	2545	38	0.027369892
B5	A>G	2545	175	0.126045553
B5	A>T	2545	185	0.133248156
B5	C>A	1259	21	0.030575307
B5	C>G	1259	9	0.013103703
B5	C>T	1259	140	0.203835382
B5	G>A	1018	79	0.14225141
B5	G>C	1018	11	0.019807158
B5	G>T	1018	30	0.054019523
B5	T>A	3802	198	0.09546196
B5	T>C	3802	275	0.132586055
B5	T>G	3802	45	0.0216959
X_r	A>C	2599	2	0.004497888
X_r	A>G	2599	71	0.159675033
X_r	A>T	2599	13	0.029236274
X_r	C>A	1338	4	0.017473859
X_r	C>G	1338	2	0.008736929
X_r	C>T	1338	76	0.332003318
X_r	G>A	1128	30	0.155452282
X_r	G>T	1128	4	0.020726971
X_r	G>C	1128	0	0
X_r	T>A	4037	24	0.03474861
X_r	T>C	4037	157	0.227313824
X_r	T>G	4037	7	0.010135011
B1_r	A>C	2599	1	0.002040154
B1_r	A>G	2599	69	0.140770628
B1_r	A>T	2599	19	0.038762927
B1_r	C>A	1338	6	0.023777401
B1_r	C>G	1338	2	0.0079258
B1_r	C>T	1338	97	0.38440131
B1_r	G>A	1128	21	0.098714155
B1_r	G>C	1128	1	0.004700674
B1_r	G>T	1128	6	0.028204044
B1_r	T>A	4035	20	0.026281836
B1_r	T>C	4035	179	0.235222429
B1_r	T>G	4035	7	0.009198642
B6	A>C	2433	3	0.00992992
B6	A>G	2433	50	0.165498664
B6	A>T	2433	12	0.039719679
B6	C>A	1430	5	0.028157919
B6	C>G	1430	1	0.005631584
B6	C>T	1430	49	0.275947611
B6	G>A	1289	24	0.14994256
B6	G>C	1289	1	0.006247607
B6	G>T	1289	5	0.031238033
B6	T>A	3919	9	0.018494127
B6	T>C	3919	125	0.256862879
B6	T>G	3919	6	0.012329418
B7	A>C	2443	2	0.007546093
B7	A>G	2443	51	0.192425366
B7	A>T	2443	13	0.049049603
B7	C>A	1430	3	0.019337522

Branch	Mutation	Count_Ref	Count_Alt	RelativeMutationRate
B7	C>G	1430	2	0.012891682
B7	C>T	1430	45	0.290062836
B7	G>A	1293	12	0.085545729
B7	G>C	1293	2	0.014257622
B7	G>T	1293	4	0.028515243
B7	T>A	3928	15	0.035199411
B7	T>C	3928	101	0.237009365
B7	T>G	3928	12	0.028159529
Hu	A>C	24548590	66	0.005672232
Hu	A>G	24548590	573	0.049245286
Hu	A>T	24548590	126	0.010828806
Hu	C>A	13561463	97	0.015090436
Hu	C>G	13561463	27	0.004200431
Hu	C>T	13561463	3211	0.499540101
Hu	G>A	11037686	404	0.077221801
Hu	G>C	11037686	70	0.013380015
Hu	G>T	11037686	1258	0.240457984
Hu	T>A	38037701	130	0.007210505
Hu	T>C	38037701	1181	0.065504665
Hu	T>G	38037701	210	0.011647739

Table. S2. Mutation spectrum (RshSTT182)

Branch	Mutation	Count_Ref	Count_Alt	RelativeMutationRate
B5	A>C	2566	41	0.026901921
B5	A>G	2566	181	0.118762137
B5	A>T	2566	188	0.123355148
B5	C>A	1294	22	0.028624934
B5	C>G	1294	12	0.0156136
B5	C>T	1294	150	0.195170001
B5	G>A	1075	119	0.186377971
B5	G>C	1075	11	0.017228216
B5	G>T	1075	33	0.051684647
B5	T>A	3912	214	0.092102413
B5	T>C	3912	291	0.125242067
B5	T>G	3912	44	0.018936945
B4	A>C	2391	27	0.024041736
B4	A>G	2391	70	0.062330428
B4	A>T	2391	188	0.167401721
B4	C>A	1179	39	0.070425907
B4	C>G	1179	5	0.009028962
B4	C>T	1179	123	0.222112475
B4	G>A	1009	85	0.179353313
B4	G>C	1009	5	0.010550195
B4	G>T	1009	20	0.04220078
B4	T>A	3666	170	0.098727492
B4	T>C	3666	174	0.101050492
B4	T>G	3666	22	0.012776499
B3	A>C	2386	23	0.019830924
B3	A>G	2386	159	0.137092043
B3	A>T	2386	65	0.056043917
B3	C>A	1212	19	0.032250507
B3	C>G	1212	2	0.00339479
B3	C>T	1212	179	0.303833722
B3	G>A	991	97	0.20136484
B3	G>C	991	7	0.014531483
B3	G>T	991	12	0.024911114
B3	T>A	3622	92	0.05225465
B3	T>C	3622	239	0.135748493
B3	T>G	3622	33	0.018743516
B2	A>C	2400	14	0.019816559
B2	A>G	2400	80	0.113237482
B2	A>T	2400	89	0.125976698
B2	C>A	1218	12	0.033469206
B2	C>G	1218	3	0.008367302
B2	C>T	1218	102	0.284488254
B2	G>A	996	60	0.204646051
B2	G>C	996	1	0.003410768
B2	G>T	996	9	0.030696908
B2	T>A	3631	87	0.081396262
B2	T>C	3631	95	0.088880976
B2	T>G	3631	6	0.005613535
B1	A>C	2361	7	0.010178177
B1	A>G	2361	65	0.094511647
B1	A>T	2361	42	0.061069064
B1	C>A	1208	8	0.022734794
B1	C>G	1208	2	0.005683698
B1	C>T	1208	139	0.395017039
B1	G>A	1012	39	0.132297628

Branch	Mutation	Count_Ref	Count_Alt	RelativeMutationRate
B1	G>C	1012	2	0.006784494
B1	G>T	1012	11	0.037314716
B1	T>A	3632	34	0.032136682
B1	T>C	3632	201	0.189984505
B1	T>G	3632	13	0.012287555
X	A>C	2338	6	0.011392426
X	A>G	2338	72	0.13670911
X	A>T	2338	34	0.06455708
X	C>A	1197	3	0.011125936
X	C>G	1197	3	0.011125936
X	C>T	1197	100	0.370864544
X	G>A	999	31	0.137754461
X	G>T	999	4	0.017774769
X	T>A	3608	37	0.045524445
X	T>C	3608	146	0.179636999
X	T>G	3608	11	0.013534294
B1_r	A>C	2608	12	0.01234703
B1_r	A>G	2608	89	0.091573804
B1_r	A>T	2608	72	0.074082178
B1_r	C>A	1371	18	0.035230912
B1_r	C>G	1371	5	0.009786364
B1_r	C>T	1371	169	0.330779117
B1_r	G>A	1133	65	0.153947372
B1_r	G>C	1133	3	0.007105263
B1_r	G>T	1133	25	0.059210528
B1_r	T>A	4050	77	0.05101813
B1_r	T>C	4050	241	0.159680121
B1_r	T>G	4050	23	0.015239182
X_r	A>C	2589	7	0.012248179
X_r	A>G	2589	67	0.11723257
X_r	A>T	2589	32	0.055991675
X_r	C>A	1360	12	0.039971263
X_r	C>G	1360	6	0.019985631
X_r	C>T	1360	102	0.339755735
X_r	G>A	1122	33	0.133237543
X_r	G>T	1122	6	0.024225008
X_r	T>A	4031	43	0.048323812
X_r	T>C	4031	168	0.188800011
X_r	T>G	4031	18	0.020228573
B7	A>C	2438	2	0.007048367
B7	A>G	2438	50	0.176209163
B7	A>T	2438	12	0.042290199
B7	C>A	1441	1	0.005962497
B7	C>G	1441	2	0.011924995
B7	C>T	1441	55	0.327937357
B7	G>A	1298	17	0.112529506
B7	G>C	1298	2	0.013238765
B7	G>T	1298	4	0.026477531
B7	T>A	3917	15	0.032902574
B7	T>C	3917	98	0.214963482
B7	T>G	3917	13	0.028515564
B6	A>C	2428	5	0.016812029
B6	A>G	2428	45	0.151308264
B6	A>T	2428	12	0.04034887
B6	C>A	1441	5	0.028327278
B6	C>G	1441	1	0.005665456
B6	C>T	1441	52	0.294603688

Branch	Mutation	Count_Ref	Count_Alt	RelativeMutationRate
B6	G>A	1294	25	0.157726457
B6	G>C	1294	1	0.006309058
B6	G>T	1294	4	0.025236233
B6	T>A	3908	10	0.02089028
B6	T>C	3908	115	0.24023822
B6	T>G	3908	6	0.012534168

Table. S3. Mutation spectrum (BANAL-52)

Branch	Mutation	Count_Ref	Count_Alt	RelativeMutationRate
B5	A>C	2721	40	0.026018145
B5	A>G	2721	193	0.125537549
B5	A>T	2721	191	0.124236642
B5	C>A	1340	22	0.029057802
B5	C>G	1340	14	0.018491329
B5	C>T	1340	158	0.208687851
B5	G>A	1075	79	0.130065917
B5	G>C	1075	11	0.018110444
B5	G>T	1075	41	0.067502564
B5	T>A	4146	229	0.09775772
B5	T>C	4146	318	0.135750895
B5	T>G	4146	44	0.018783143
B4	A>C	2769	17	0.013262265
B4	A>G	2769	113	0.088155054
B4	A>T	2769	223	0.173969709
B4	C>A	1357	23	0.036613371
B4	C>G	1357	5	0.007959429
B4	C>T	1357	157	0.249926055
B4	G>A	1104	96	0.187842513
B4	G>C	1104	3	0.005870079
B4	G>T	1104	25	0.048917321
B4	T>A	4194	198	0.101983167
B4	T>C	4194	144	0.074169576
B4	T>G	4194	22	0.011331463
B3	A>C	2711	29	0.023859776
B3	A>G	2711	179	0.147272413
B3	A>T	2711	83	0.068288326
B3	C>A	1330	24	0.040249222
B3	C>G	1330	4	0.006708204
B3	C>T	1330	126	0.211308416
B3	G>A	1105	110	0.222038507
B3	G>C	1105	10	0.020185319
B3	G>T	1105	10	0.020185319
B3	T>A	4221	130	0.068695121
B3	T>C	4221	290	0.153242961
B3	T>G	4221	34	0.017966416
B2	A>C	2733	14	0.013926198
B2	A>G	2733	129	0.128319963
B2	A>T	2733	112	0.11140958
B2	C>A	1336	17	0.034592871
B2	C>G	1336	5	0.010174374
B2	C>T	1336	133	0.270638345
B2	G>A	1120	75	0.182048618
B2	G>C	1120	2	0.00485463
B2	G>T	1120	13	0.031555094
B2	T>A	4235	114	0.073180536
B2	T>C	4235	205	0.131596577
B2	T>G	4235	12	0.007703214
X	A>C	2636	2	0.004956688
X	A>G	2636	50	0.123917203
X	A>T	2636	12	0.029740129
X	C>A	1388	3	0.014120133
X	C>G	1388	1	0.004706711
X	C>T	1388	82	0.385950307
X	G>A	1153	22	0.124652324

Branch	Mutation	Count_Ref	Count_Alt	RelativeMutationRate
X	G>T	1153	5	0.028330074
X	T>A	4123	23	0.03644362
X	T>C	4123	150	0.237675781
X	T>G	4123	6	0.009507031
B1	A>C	2670	4	0.01021068
B1	A>G	2670	48	0.122528165
B1	A>T	2670	13	0.033184711
B1	C>A	1399	4	0.019487146
B1	C>G	1399	1	0.004871786
B1	C>T	1399	76	0.370255767
B1	G>A	1167	18	0.105125386
B1	G>T	1167	8	0.046722394
B1	T>A	4147	22	0.036157184
B1	T>C	4147	145	0.238308713
B1	T>G	4147	8	0.013148067
B7	A>C	2443	2	0.007561494
B7	A>G	2443	52	0.19659885
B7	A>T	2443	13	0.049149712
B7	C>A	1438	2	0.012846127
B7	C>G	1438	1	0.006423063
B7	C>T	1438	47	0.301883981
B7	G>A	1295	14	0.099852597
B7	G>C	1295	2	0.014264657
B7	G>T	1295	4	0.028529313
B7	T>A	3918	15	0.035361276
B7	T>C	3918	93	0.219239909
B7	T>G	3918	12	0.028289021
B6	A>C	2433	4	0.012778658
B6	A>G	2433	48	0.153343895
B6	A>T	2433	12	0.038335974
B6	C>A	1438	5	0.027025795
B6	C>G	1438	1	0.005405159
B6	C>T	1438	57	0.308094064
B6	G>A	1291	23	0.138474229
B6	G>C	1291	2	0.012041237
B6	G>T	1291	5	0.030103093
B6	T>A	3909	9	0.017895515
B6	T>C	3909	123	0.244572038
B6	T>G	3909	6	0.011930343
X_r	A>C	2585	1	0.002823844
X_r	A>G	2585	46	0.129896831
X_r	A>T	2585	11	0.031062286
X_r	C>A	1343	3	0.016305965
X_r	C>T	1343	66	0.358731238
X_r	G>A	1142	21	0.134231506
X_r	G>T	1142	3	0.019175929
X_r	T>A	4032	20	0.036208518
X_r	T>C	4032	140	0.253459623
X_r	T>G	4032	10	0.018104259
B1_r	A>C	2620	3	0.007807712
B1_r	A>G	2620	42	0.109307963
B1_r	A>T	2620	10	0.026025706
B1_r	C>A	1354	5	0.025179966
B1_r	C>G	1354	1	0.005035993
B1_r	C>T	1354	71	0.357555521
B1_r	G>A	1158	20	0.117767441
B1_r	G>C	1158	2	0.011776744

Branch	Mutation	Count_Ref	Count_Alt	RelativeMutationRate
B1_r	G>T	1158	6	0.035330232
B1_r	T>A	4057	19	0.031933932
B1_r	T>C	4057	153	0.25715219
B1_r	T>G	4057	9	0.015126599

Data availability

The source data are all publically available. The intermediate data supporting the findings of the present study are included as Supplementary Datasets I-VII.

Code availability

The related codes are available at https://github.com/shadowdeng1994/SarsMutSignature.

References for Supporting Information

11 Shan, K. J., Wei, C., Wang, Y., Huan, Q. & Qian, W. Host-specific asymmetric accumulation of mutation types reveals that the origin of SARS-CoV-2 is consistent with a natural process. *Innovation (N Y)* **2**, 100159, doi:10.1016/j.xinn.2021.100159 (2021).

12 Slater, G. S. & Birney, E. Automated generation of heuristics for biological sequence comparison. *BMC Bioinformatics* **6**, 31, doi:10.1186/1471-2105-6-31 (2005).

13 Elbe, S. & Buckland-Merrett, G. Data, disease and diplomacy: GISAID's innovative contribution to global health. *Glob Chall* **1**, 33-46, doi:10.1002/gch2.1018 (2017).

14 Abascal, F., Zardoya, R. & Telford, M. J. TranslatorX: multiple alignment of nucleotide sequences guided by amino acid translations. *Nucleic Acids Res* **38**, W7-13, doi:10.1093/nar/gkq291 (2010).

15 Katoh, K. & Standley, D. M. MAFFT multiple sequence alignment software version 7: improvements in performance and usability. *Mol Biol Evol* **30**, 772-780, doi:10.1093/molbev/mst010 (2013).

16 Borowiec, M. L. AMAS: a fast tool for alignment manipulation and computing of summary statistics. *PeerJ* **4**, e1660, doi:10.7717/peerj.1660 (2016).

17 Nguyen, L. T., Schmidt, H. A., von Haeseler, A. & Minh, B. Q. IQ-TREE: a fast and effective stochastic algorithm for estimating maximum-likelihood phylogenies. *Mol Biol Evol* **32**, 268-274, doi:10.1093/molbev/msu300 (2015).

18 Kumar, S., Stecher, G., Li, M., Knyaz, C. & Tamura, K. MEGA X: Molecular Evolutionary Genetics Analysis across Computing Platforms. *Mol Biol Evol* **35**, 1547-1549, doi:10.1093/molbev/msy096 (2018).

19 Hul, V. *et al.* A novel SARS-CoV-2 related coronavirus in bats from Cambodia. *BioRxiv*, doi:https://doi.org/10.1101/2021.01.26.428212 (2021).
